# Synergistic Antibacterial Effects of Metallic Nanoparticle Combinations

**DOI:** 10.1038/s41598-019-52473-2

**Published:** 2019-11-05

**Authors:** C. Bankier, R. K. Matharu, Y. K. Cheong, G. G. Ren, E. Cloutman-Green, L. Ciric

**Affiliations:** 1 Department of Civil, Environmental and Geomatic Engineering, University College London, London, WC1E 7JE UK; 2 Department of Mechanical Engineering, University College London, London, WC1E 7JE UK; 3 School of Engineering and Technology, University of Hertfordshire, Hatfield, AL10 9AB UK; 4 Department of Microbiology, Virology, and Infection Prevention Control, Great Ormond Street Hospital NHS Foundation Trust, London, WCIN 3JH UK

**Keywords:** Antibiotics, Nanoparticles

## Abstract

Metallic nanoparticles have unique antimicrobial properties that make them suitable for use within medical and pharmaceutical devices to prevent the spread of infection in healthcare. The use of nanoparticles in healthcare is on the increase with silver being used in many devices. However, not all metallic nanoparticles can target and kill all disease-causing bacteria. To overcome this, a combination of several different metallic nanoparticles were used in this study to compare effects of multiple metallic nanoparticles when in combination than when used singly, as single elemental nanoparticles (SENPs), against two common hospital acquired pathogens (*Staphylococcus aureus* and *Pseudomonas. aeruginosa*). Flow cytometry LIVE/DEAD assay was used to determine rates of cell death within a bacterial population when exposed to the nanoparticles. Results were analysed using linear models to compare effectiveness of three different metallic nanoparticles, tungsten carbide (WC), silver (Ag) and copper (Cu), in combination and separately. Results show that when the nanoparticles are placed in combination (NPCs), antimicrobial effects significantly increase than when compared with SENPs (P < 0.01). This study demonstrates that certain metallic nanoparticles can be used in combination to improve the antimicrobial efficiency in destroying morphologically distinct pathogens within the healthcare and pharmaceutical industry.

## Introduction

Nanotechnology has played a crucial role in the advancement of a variety of fields. In particular, nanoparticles (NPs) have been widely utilised in several industries, ranging from biomedicine to engineering, due to their unique size-dependent physical and chemical properties (e.g. high surface to volume ratio)^1^. One example is the use of biodegradable NPs for drug delivery whereby the active ingredient can adsorb or absorb to the NP which can then deliver it to targeted areas^2^. Furthermore, several metallic NPs are widely accepted as having antimicrobial properties with many, in particular silver, now being used in medical devices to help decontaminate equipment and prevent the spread of infectious disease^3^. More recently, metallic NPs have been used in combination with certain antibiotics to help overcome resistant bacteria and enhance antibiotic effects^4,5^. However, these therapies are designed to be used once symptoms of infection manifest and certain metallic NPs can be toxic to mammalian cells when used internally as therapeutics^6^.

Industry and healthcare are keen to develop new ways to prevent and control infection by using materials that target a broad-spectrum of microbes whilst remaining non-toxic to human cells when used indirectly in patient care (i.e. medical devices, surfaces and manufacturing of drugs). NPs could offer a novel way to reduce the cost and use of multiple disinfectants and procedures by integrating these antimicrobial particles into clinical and industrial devices. Multiple studies have shown strong antimicrobial effects of metallic NPs to multiple species of bacteria^7–11^. However, it has also been shown that although certain NPs might be effective antimicrobials against certain bacteria, they may have little or no effect on others. Ideally, when using NPs integrated into devices to prevent contamination, a broad spectrum NP would be used to target multiple species of bacteria. Using a combination of metallic NPs that can target a broad-spectrum of pathogens could be an effective solution for devices used in healthcare.

Different NPs employ different mechanisms to destroy bacteria and in particular metallic NPs are known to use several modes of action to do this. NPs have been shown to penetrate the bacterial cell wall and form pores on the surface of the membrane which, in turn, causes free radical formation which can also destroy the cell membrane^12^. Ions from the NPs can interfere with enzyme production and generate reactive oxygen species (ROS). DNA transcription has also been shown to be affected^13^.

*Staphylococcus aureus* and *Pseudomonas aeruginosa* are two pathogens of concern to the healthcare and pharmaceutical industry as they are highly resistant to antibiotics^14,15^. In healthcare, it is common for *P. aeruginosa* to form biofilms in equipment which can lead to respiratory infections like ventilator associated pneumonia in immunocompromised patients^16^. *S. aureus* and *P. aeruginosa* are known to cause critical illness through skin, ear and eye infections and are notoriously difficult to treat due to resistance to numerous therapies^14^. Many studies have shown antimicrobial effects of single metallic NPs on these pathogens and therefore, by integrating different combinations of known antimicrobial metallic NPs (such as aluminimum oxide, zinc oxide, titanium dioxide, nickel oxide and silver) within devices at specific concentrations could prevent contamination by these pathogens^8,17–19^.

In this study, the effects of single elemental nanoparticles (SENPs) - silver, copper and tungsten carbide - on Gram-positive (*S. aureus*) and Gram-negative (*P. aeruginosa*) bacteria were analysed. Silver and copper NPs were chosen for this study as they are common antibacterial agents, tungsten carbide was also chosen as though the antiviral properties of this material have been previously reported, the antibacterial properties are unknown^20,21^. Further to this, the effects of a combinations of nanoparticles (NPCs) of the same elements together in different ratios were investigated on the same bacterial species at various concentrations (0.05, 0.10 and 0.25 w/v %) to determine the strongest antibacterial effect. The effects of the SENPs and NPCs on both species have been assessed by quantifying the proportions of live and dead cells after exposure to these NP treatments after 24 hours. Quantification of bacterial cells was performed using flow cytometry and the synergistic effects of NPCs were compared to SENPs.

## Results

To determine the antibacterial effect of SENPs and NPCs on *P. aeruginosa* and *S. aureus*, populations of live and dead bacteria were quantified using flow cytometry after treatment.

### Single elemental nanoparticles (SENP)

**Figure 1:**
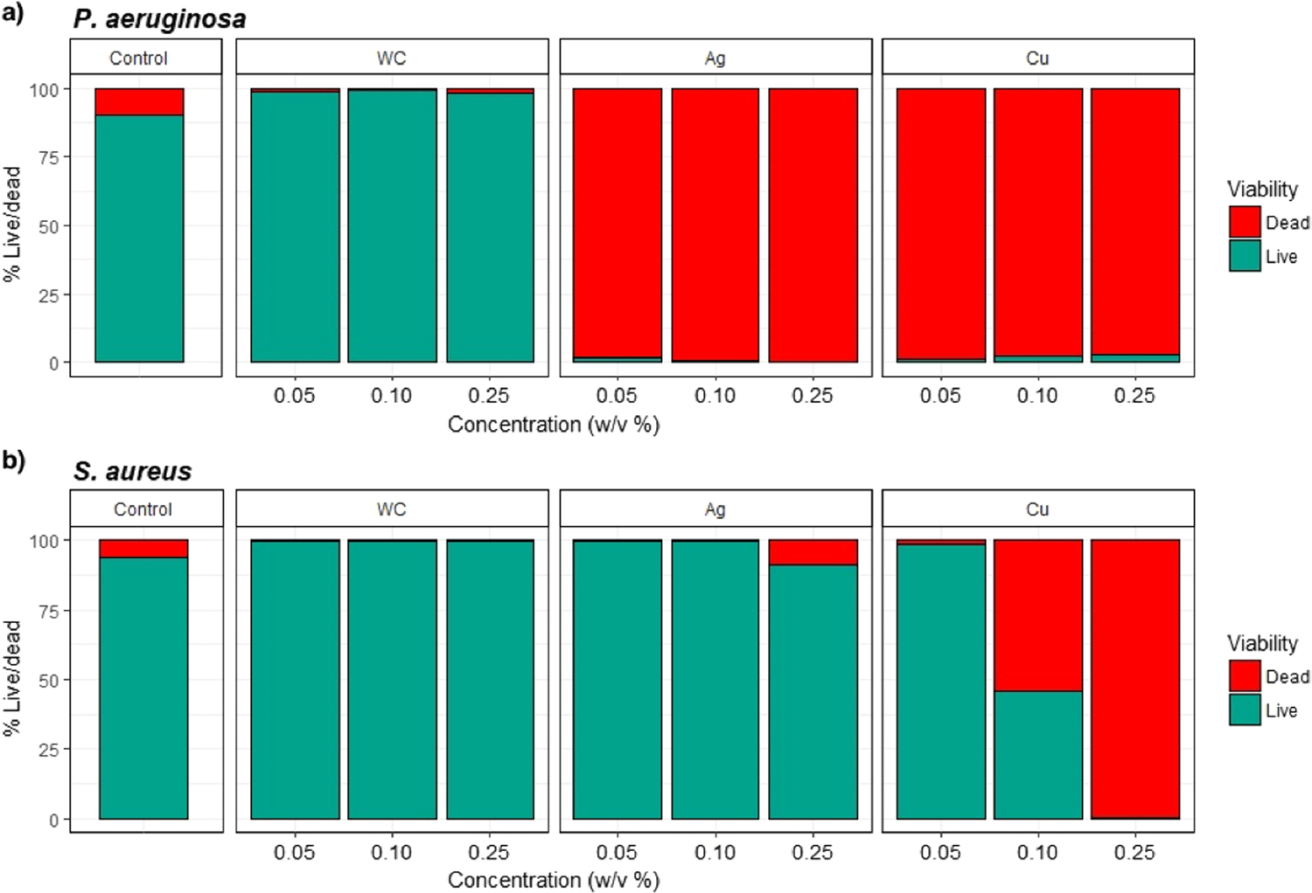
Exposure of (**a**) *P. aeruginosa* and (**b**) *S. aureus* to single elemental nanoparticles (SENPs). Green shows live cells and red shows proportion of dead cells (*n* = 3).

Figure 1 shows the results obtained when *S. aureus* and *P. aeruginosa* were exposed to SENPs for 24 hours. In this figure the control samples represent the bacterial population prior to SENP exposure. The green bars show the proportion of ‘live’ cells whilst the red bars symbolise the proportion of ‘dead’ cells in suspension after incubation. From these results it can be concluded that WC did not exhibit any antibacterial activity against *P. aeruginosa* as a large proportion of the cells were still alive (∼98% ± 0.38). However, a strong antimicrobial effect was observed for all concentrations of Ag (>99% dead) and Cu with most cells showing damaged membranes (>98%). Furthermore, it was also noted that the antibacterial activity of Ag and Cu NPs against *P. aeruginosa* was not dose dependent.

Similarly, WC NPs did not show any antimicrobial effect on *S. aureus* (>99% ± 0.14 live), whereas Ag NPs displayed some mild cytotoxic activity at higher concentrations (9% ± 0.9 dead at 0.25 w/v %) but this was still not as effective as it was on *P. aeruginosa*. Cu NPs showed antimicrobial properties towards *S. aureus* cells at higher concentrations (at 0.10 w/v%, 55%, ±3.4 of the cells were dead, whilst at 0.25 w/v %, ~99% ± 0.08 of the tested population was dead), therefore indicating antimicrobial activity was dose dependent.

A one-way ANOVA showed a significant difference between proportions of live and dead cells for *P. aeruginosa* (F_3,26_ = 17025, P = 0.0001) with *post hoc* TukeyHSD showing significant differences between all nanoparticles (P = 0.0001) with the exception of Ag compared with Cu (P = 0.155).

Similarly, for *S. aureus*, there was also an overall significant difference between live and dead cells (F_3,26_ = 9.012, P = 0.0002). TukeysHSD shows a significant difference between Cu with the control, WC and Ag (P < 0.001) with no significant difference shown between WC, control or Ag (P = 0.9).

### Nanoparticle combinations (NPC)

**Figure 2:**
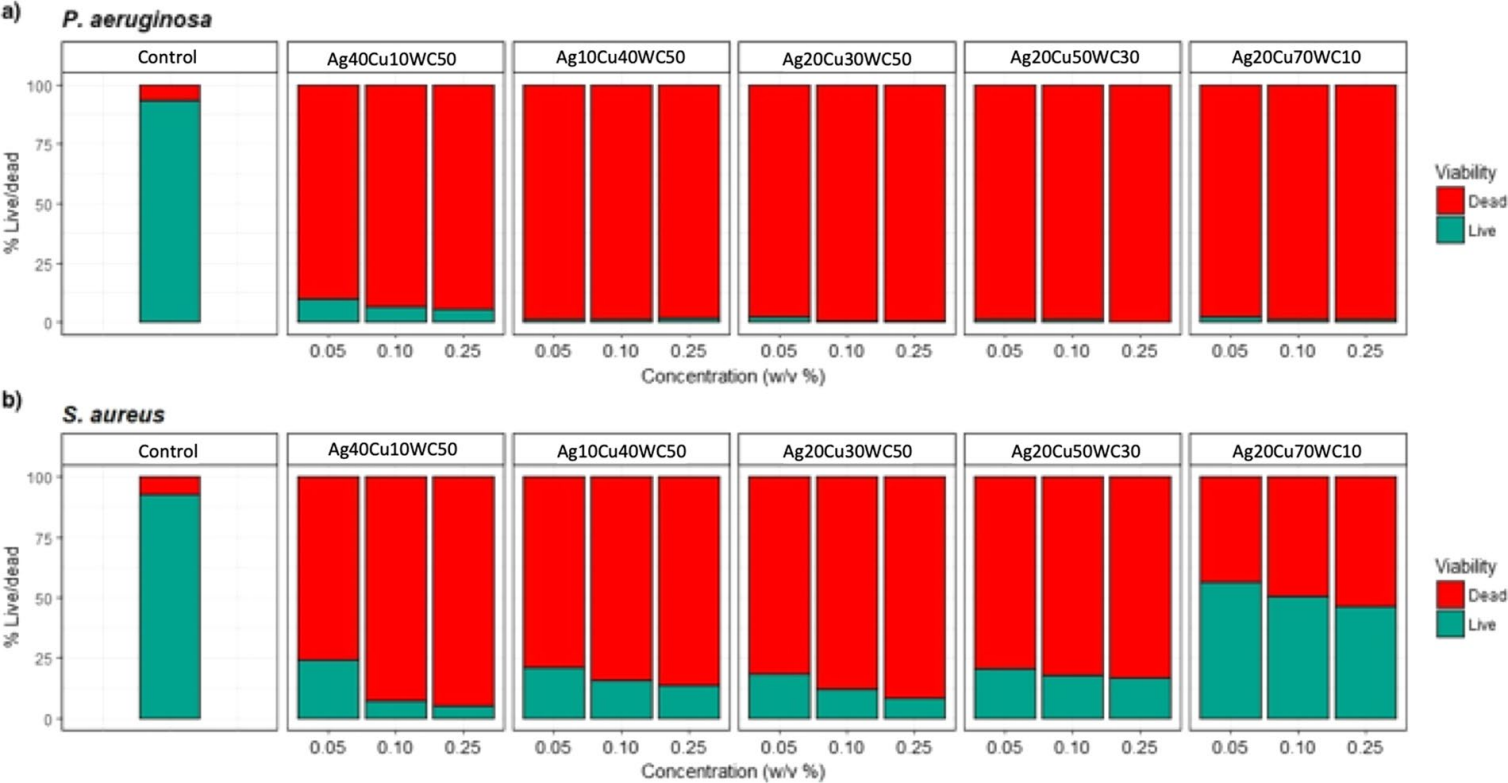
Exposure of (**a**) *P. aeruginosa* and (**b**) *S. aureus* to nanoparticle combinations (NPCs). Green shows live cells and red shows proportion of dead cells (*n* = 3).

To determine the antimicrobial effect of the SENPs when in combination (NPCs), populations of live and dead bacterial cells were analysed after exposure (Fig. 2). The NPCs were named as shown in Table 1.

**Table 1:** Composition of Antimicrobial Nanoparticle Formulations.

Nanocomposite Formulation	Weight Ratio of Each Nanoparticle Component (w/w%)		
	Silver	Copper	Tungsten Carbide
Ag	100	—	—
Cu	—	100	—
WC	—	—	100
Ag40Cu10WC50	40	10	50
Ag10Cu40WC50	10	40	50
Ag20Cu30WC50	20	30	50
Ag20Cu50WC30	20	50	30
Ag20Cu70WC10	20	70	10

Antimicrobial effects of NPCs were most pronounced against *P. aeruginosa* with all NPCs showing cell death at >90% for all concentrations. All NPCs, other than Ag20Cu70WC10, were most effective on *S. aureus* with >80% killing rate after exposure. However, Ag20Cu70WC10 did not show as potent an effect with only 40% ±0.55 cell death at the lowest concentration (0.05 w/v %) and increasing to ∼50% ± 0.71 at the highest concentration (0.25 w/v %).

A one-way ANOVA revealed a significant difference overall for *P. aeruginosa* and *S. aureus* for all treatments (F_5,10_ = 1141, P = 0.0001. F_5,10_ = 44.2, P = 0.0001, respectively). The *post hoc* TukeyHSD for *P. aeruginosa* data, significant differences were shown for Ag40Cu10WC50 and all other combinations of particles and the control (P < 0.001). There was no significance between Ag10Cu40WC50, Ag20Cu30WC50, Ag20Cu50WC30 and Ag20Cu70WC10 (P > 0.92).

Similar results are shown for *S. aureus*, however, the antimicrobial effect is less evident with some cells surviving after exposure to treatment (green bars), in particular, Ag20Cu70WC10. *Post hoc* TukeyHSD shows a significant difference between the controls and NPCs (P < 0.0001). However, no significant difference was shown between Ag40Cu10WC50, Ag10Cu40WC50, Ag20Cu30WC50 and Ag20Cu50WC30 (P > 0.80). Ag20Cu70WC10 treatment was significantly less effective when compared with all other treatments (P < 0.0001).

For *S. aureus*, there is a trend of increasing cell death as concentrations of NPCs increase (from 0.05 to 0.25 w/v %) but antimicrobial effects are not as strong when compared to *P. aeruginosa*. It was also noted that, at higher concentrations, the NPCs were more toxic towards *P. aeruginosa*, as a stronger kill was observed.

### Single elemental nanoparticles vs nanoparticle combinations

**Figure 3:**
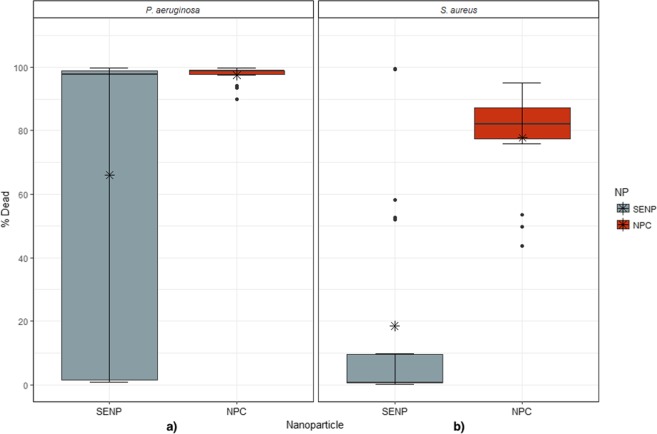
Boxplot showing mean % cell death for all SENP and NPCs for (**a**) *P. aeruginosa* and (**b**) *S. aureus*. The top and bottom boundaries of each box indicate upper and lower quartile values, and black horizontal lines inside each box represent the median. Ends of the whiskers mark the lowest and highest % cell death observed from each treatment. Asterisk represent the mean and dots show outliers.

To help visualise the potential synergistic antimicrobial effects of each SENP (WC, Ag and Cu) and NPCs, the mean of each SENP and NPC tested was calculated for all concentrations (Fig. 3). Results show that, for *P. aeruginosa*, there is a significant variation in the proportion of dead cells between SENP and NPC, but this remained relatively consistent across all NPCs tested (one-way ANOVA: F_1,40_ = 6.6, P = 0.01). Similarly, for *S. aureus*, there was a significant difference in cell death rates between SENP and NPC (one-way ANOVA: F_1,40_ = 41, P = 0.0001). SENP treatment showed low percentages of dead cells (0–10%) whilst NPC showed a significantly higher rate of cell death of between ∼77 and 88%.

## Discussion

Various metallic NPs are widely known to have antimicrobial effects, however, not all metallic NPs can destroy all types of bacteria. Therefore, when integrating NPs into healthcare or pharmaceutical devices, it is desirable to use a combination of NPs to target a broad-spectrum of pathogens. Silver and copper NPs have been used for decades as antimicrobials in multiple devices, however, implementation of these NPs into modern devices has been slow, possibly due to the specificity of the NPs to certain bacteria^22^.

Herein, the antimicrobial effect of SENPs at three different concentrations (0.05, 0.10 and 0.25 w/v %) were compared in two known distinctively different pathogens (Gram-positive *S. aureus* and Gram-negative *P. aeruginosa*). The SENPs were then combined together in different ratios and their antimicrobial effect tested on the two pathogens.

Results showed Ag SENPs had strong antibacterial effects on *P. aeruginosa* and no antibacterial effects on *S. aureus*, Cu SENPs had antimicrobial effects on *P. aeruginosa* and moderate cytotoxicity against *S. aureus* whereas WC NPs did not show any effect on the bacterial cells (Fig. 1). It was also noted that the antimicrobial effects of Ag (at all concentrations) and Cu (at 0.05 and 0.10 w/v%) SENPs were less pronounced against *S. aureus*.

The Ag SENPs used in this study exhibited strong antibacterial activity. In this research, when minimum dose of 0.05 w/v% (which equates to 0.5 mg/mL) was used against *P. aeruginosa*, 99% of the bacterial population was killed. This concentration is significnaly lower, when compared to the minimum inhibitory concentration reported by Punjabi *et al*. for the same bacteria strain (2.5 mg/mL)^23^. However, similarly, Punjabi *et al*. also noted that a higher nanoparticle concentration (5 mg/mL) is required in order to be effective against *S. aureus*^23^. This was also observed in the presented study, as a Ag SENP concentration of 0.25 w/v% only resulted in moderate cytotoxicity. The antibacterial activity of Ag SENPs has been attributed to three distinct mechanisms: firstly, it is thought that the Ag NPs bind to the surface of the cell membrane thus altering basic cellular functions including permeability and respiration (production of reactive oxygen species); secondly, it has also been reported that Ag NPs penetrate inside the bacterial cell and cause damage by interacting with sulfur- and phosphorus- containing compounds such as DNA; thirdly, studies have shown the Ag NPs to release Ag ions which then deposit in the cell and along the cell wall as granules, consequently inhibiting cell division, damaging the integtrity of the cell membrane and wall and interefering with the cellular content^24–31^. It is plausible that all three destructive mechanisms work simulutanously to cause microbial death.

In the case of Cu NPs, the antibacterial mode of action is thought to be multifaceted, whereby the release of Cu ions is the primary cytotoxic mechanism^27,32,33^. The Cu ions interact either directly with the cellular membrane or intracellulary to produce free-radicals. Alternative hypothetical mechanisms include the accumulation and dissolution of NPs in the bacterial membrane changing its permeability, with subsequent release of intracellular biomolecules and dissipation of the proton motive force across the plasma membrane^27,34–36^.

When using the NPs in combination (NPCs composition displayed in Table 1), a more pronounced antimicrobial effect was observed for both bacterial species with a high cell death rate for *P. aeruginosa* and *S. aureus* (Ag40Cu10WC50, Ag10Cu40WC50, Ag20Cu30WC50 and Ag20Cu50WC30) (Fig. 2). This was then corroborated further by showing that on average, NPCs can significantly improve the antimicrobial performance when compared to SENPs (Fig. 3).

This more pronounced antimicrobial effect is thought to be due to the ratios of NPs within each combination (Table 1). As a high cell death rate was observed for *P. aeruginosa* for all NPCs, it can be assumed that this is attributed to the Ag NP (Ag, Fig. 1) which were effective at killing *P. aeruginosa* cells in the SENP form. However, for *S. aureus*, the reason for the higher rate of cell death when NPs are in combination than when single elements is less clear. Ag20Cu70WC10, which did not have a strong antimicrobial effect, showed to have the highest quantity of copper with a small amount of silver. It is thought that copper nanoparticles might cause pores to form in Gram-positive *S. aureus* cell membrane which could allow silver particles to penetrate and destroy the cell. It has been shown previously that this type of mechanism can cause damage to cell membranes whilst being unrelated to oxidative stress^37^. As cell membranes act as important defence barriers for bacteria, the mechanism in which NPs are absorbed differs between Gram-positive and Gram-negative organisms which could explain the differences shown here. Gram-positive bacteria have a thicker (multi-layered) peptidoglycan exterior wall, functionalised with teichoic acid and tend to be more porous^38^. Whereas, Gram-negative bacteria have a single peptidoglycan layer followed by an outer membrane that is densely populated with lipopolysaccharides. However, although these physiological factors might contribute to susceptibility to antimicrobials, here, a reduced antimicrobial effect for Gram-positive than Gram-negative bacteria was observed. Similar results were shown in a study by Kim *et al*., (2007), which demonstrated Ag NPs to be effective against Gram-negative *E. coli* but not Gram-positive *S. aureus*^26^. They concluded this was due to a difference in cell wall structure between the two pathogens and type of NP used^26^. However, cell wall structure alone does not fully explain the potential for a NP to be antimicrobial. Several physico-chemical factors can contribute to the antimicrobial potential of a nanoparticle, such as size, shape and charge. Gram-positive organisms tend to attract positively charged ionic NPs due to their negatively charged cell wall, however, not all NPs are positively charged^19^. The size and shape of NPs can significantly affect their bactericidal properties. A smooth surface area can increase the potential for contact with a bacterial cell. The NPs tested here were tested in solution (LB broth), which could cause the particles to coagulate or reduce their stability which might reduce potency towards *S. aureus*, as suggested by Pal *et al*., (2007) when testing silver NPs in broth against *E. coli*^39^. A recent study by Garza-Cervantes *et al*. (2017) has shown that Cu, Zn and Ni may all increase prokaryotic cell memberane permeability when combined with Ag NPs^11^. The mode of action of metallic antimicrobial NPs on microbial cells is still very much under debate and rests on little data and many hypotheses as outlined by Shaikh *et al.* (2019) in a recent review of the litarature^40^.

Tungsten carbide (WC) was not shown to have any antimicrobial effect on either *S. aureus* or *P. aeruginosa*. For SENP, no antimicrobial effect was shown and therefore it is not thought that tungsten contributed to any antimicrobial impact on the bacterial cells when placed in combination (NPCs). It has previously been shown that tungsten does have antimicrobial effects against *S. aureus* caused by reactive oxygen species (ROS) when the NPs were shown to be <10 nm^41^. However, in this study, the WC used was larger (10–20 nm) and therefore, we believe this might have been one factor that resulted in a lower antimicrobial impact.

In summary, metallic NPs were more effective when used in combination against *S. aureus* rather than alone and very potent antimicrobial effects were shown against *P. aeruginosa* when testing both SENP and NPC. This study shows the potential for a synergistic effect of NPs when placed in combination rather than using a single NP and for combinations to target a broad spectrum of both Gram-positive and Gram-negative bacteria. The data presented here give and overall assessment of the antimicrobial effect of NPs when in combination rather than on their own on two morphologically distinct pathogens. This work could be useful to healthcare practitioners and engineers in the development of medical devices that target a wide range of bacterial pathogens.

## Materials and Methods

### Nanoparticle preparation

Tungsten carbide (WC) and silver (Ag) NPs were engineered by Intrinsig Materials® (Farnborough, UK) using Tesima^TM^ thermal plasma patented technology^21^. WC NPs were hexagonal in shape with an average diameter of 250 nm^42^. Dispersed WC NP aggregation suspenson at concentration 0.1 wt/v% showed to have hydrodynamic sizes ranging from 180–220 nm with zeta surface charge of −21.7 kv at pH 6.6. The Ag NPs were rod shaped and had particle sizes ranging from 80–90 nm in one dimension^43^. Dispersed Ag NP aggregation suspension at concentration 0.1 wt/v% showed to have hydrodynamic sizes ranging from 180–190 nm with zeta surface charge of −30.6 kv at pH 7.0. Copper (Cu) NPs were manufactured by Canfuo Nanotechnology® (Suzhou, China) and were 10–20 nm in diamter. The size information of the nanoparticles were provided by the manufacturing company. Dispersed Cu NP aggregation suspension at concentration 0.1 wt/v% showed to have hydrodynamic sizes ranging from 180–190 nm with zeta surface charge of −8.82 kv at pH 6.6. Saline solution (0.9 w/v% NaCl) was purchased from Fisher Scientific (Loughborough, England) and used as received.

For *in vitro* antimicrobial tests, antimicrobial nanoparticle formulations were prepared by suspending 1% wt/v of nano-powder composites (Table 1) into saline solution to collectively create the NPCs. In the case of Ag, 1 ppm of ammonium solution (Fisher Schientific, UK) was added to aid forming uniform distribution of silver nanoparticles suspension. Each of these suspension were sonicated using a 750 W high-frequency ultrasonic probe (Sonics & Materials®, USA) with an applied ON/OFF pulse programme and 53% working power, mixtures were sonicated for 2–3 minutes until a well dispersed suspension was formed.

### Growth of bacteria

Stock cultures of bacterial strains *P. aeruginosa* NCTC 12903 *and S. aureus* ATCC 6538 P were stored in 30% glycerol. The strains were cultured on Tryptic Soya Agar (TSA, Sigma, England) and the plates were incubated at 37 °C for 24 hours. After incubation, a single colony of each bacterial strain was picked using a sterile loop and grown in 20 mL of Lennox I Broth (LB, Invitrogen, England) at 37 °C with constant agitation at 150 rpm for a further 24 hours. The bacterial cultures were then diluted 1:100 (equivalent to a cfu of 10^8^) and inoculated into the NP preparations (SENP and NPCs) at three different concentrations (0.05, 0.10 and 0.25 w/v %) in LB, in triplicate and placed on a shaker for a further 24 hours. Controls included a negative control of LB only and NPs only, and a positive control of bacteria with no NP treatments.

### Flow cytometry

To quantify the effects of the NPs on the bacterial populations, flow cytometry was used in conjuction with the LIVE/DEAD BacLight Bacterial Viability assay (ThermoFisher), to determine the proportion of live and dead cells after incubation with NPs. The fundamental principle of the assay relies on fluorescent dyes, Propidium Iodide (PI) and SYTO®9, to stain live and dead cell populations. PI is a red fluorescent interclating stain which penetrates cells with damaged membranes, whilst SYTO®9 is a green fluorescent nucleic stain which is able to penetrate both live and dead cells and bind to the nucleic acid. When both dyes are present, PI exhibits a stronger affinity for nucleic acids than SYTO®9, and hence, SYTO®9 is displaced by PI and a distinction can be made between live and dead cells^44^. A stock solution containing both dyes was prepared according to manufacturer’s recommendations. The staining solution was added to a 1:1000 dilution of the bacteria treated with SENPs and NPCs in microcentrifuge tubes, in triplicate with appropriate controls. After the stain was added, samples were incubated in the dark at room temperature for 15 minutes.

**Figure 4:**
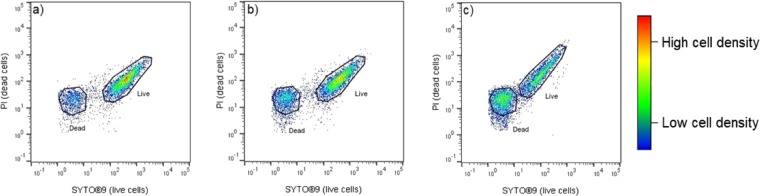
Gating strategy example of *S. aureus* bacterial cells after exposure to Ag20Cu70WC10 as acquired by flow cytometry. Events are visualised as a density plot and gated using FlowJo V10 (Treestar, USA). (**a**) Live and dead proportion of cells after exposure to 0.05 w/v% nanoparticle combinations, (**b**) after exposure to 0.10 w/v % and (**c**) 0.25 w/v %. An increase in the number of dead cells and an increase in live is shown as concentration of Ag20Cu70WC10 increases. Events outside of gating (black lines) are excluded from the analysis as these are indeterminate events.

Post incubation, cells were acquired using a calibrated Guava easyCyte® flow cytometer (Merck, UK) using InCyte software (Merck, UK). Gates were set up accordingly using positive (bacteria only), negative (media only) and fluorescent minus one (FMO) controls (single stained positive controls). 50,000 events were collected overall and bacteria acquisition gates were determined using forward scatter and side scatter channels to eliminate background noise and debris from the sample. The gated population of bacteria was then analysed using fluorescent channels FL1 (live populations, SYTO®9) vs FL3 (dead populations, PI), as shown in Fig. 4.

FlowJo (V10, TreeStar, USA) was used to gate the live and dead bacterial cell counts where proportions of live and dead bacteria cells were calculated.

### Statistical analysis

All analyses were performed using RStudio (v 1.0.136, USA), software with graphics coded via the ggplot2 package. The Shapiro-Wilk test was used to check the data for normality and subsequently a one-way ANOVA with *post hoc* TukeyHSD were performed. Significant difference was defined as P < 0.05.

## Data Availability

All data is presented in the paper.
